# Antifungal effect of the bark and root extracts of *Punica granatum* on oral *Candida* isolates

**DOI:** 10.18502/cmm.4.4.382

**Published:** 2018-12

**Authors:** Fatemeh Lavaee, Darya Motaghi, Amir Reza Jassbi, Hadis Jafarian, Fatemeh Ghasemi, Parisa Badiee

**Affiliations:** 1 Department of Oral and Maxillofacial Medicine, Oral and Dental Disease Research Center, School of Dentistry, Shiraz University of Medical Sciences, Shiraz, Iran; 2 Student Research Committee, School of Dentistry, Shiraz University of Medical Sciences, Shiraz, Iran; 3 Medicinal and Natural Products Chemistry Research Center, Shiraz University of Medical Sciences, Shiraz, Iran; 4 Alborzi Clinical Microbiology Research Center, Shiraz University of Medical Sciences, Shiraz, Iran

**Keywords:** Candida, Fluconazole, Liver transplantation, Nystatin, Punica granatum

## Abstract

**Background and Purpose:**

Oral candidiasis is one of the most common fungal infections in humans. The treatment and prophylaxis of the patients suffering from this infection require the identification of new anti-*Candida* agents with no side effects or toxicity like medicinal plants. The present study was conducted to compare the antifungal activities of the aqueous, ethanolic, and methanolic extracts of the bark and roots of *P. granatum* with those of two routine antifungal agents (i.e., fluconazole and nystatin) on oral *Candida *strains isolated from liver transplant recipients.

**Materials and Methods:**

Minimum inhibitory concentrations (MICs) of the ethanolic, methanolic, and aqueous extracts of the bark and root of *Punica granatum* against *C. albicans* and *C. glabrata *isolated from oral cavities were evaluated according to the CLSI M27-A3. All data were analyzed in SPSS (version 16.0) by pairwise comparison and Kruskal-Wallis test.

**Results:**

The MIC50 and MIC90 values for the methanolic and ethanolic extracts of the bark and root of *P. granatum *against *C. albicans* were both obtained as 0.05 mg/ml with the geometric mean (GM) of 0.07. Furthermore, the MIC90 values for the aqueous extracts of bark and root were estimated as 0.05 and 0.2 mg/ml, respectively. With regard to *C. glabrata,* the MIC50 and MIC90 values for the methanolic and ethanolic extracts of the bark and root were 0.05 mg/ml. However, the MIC90 value for the aqueous extract against this species was obtained as 25 mg/ml. The GM values for the aqueous extracts of the bark and root were 9.49 and 0.32, respectively.

**Conclusion:**

As the findings indicated, the methanolic and ethanolic extracts of the bark and root of *Punica granatum* had anti-*Candida* activities. Therefore, they can be considered as mouthwash or toothpaste to prevent and treat *Candida* infections in the oral cavity.

## Introduction

Oral candidiasis is an opportunistic infection of the oral cavity. Infection is common among the elderly, especially in those with dentures [1]. *Candida* species are saprophytic yeasts and normal flora of the oral cavity, vaginal mucosa, and gastrointestinal tract. However, they can cause systemic diseases in immunocompromised patients, such as those with HIV, organ or bone marrow transplantation, under chemotherapy or radiotherapy, and diabetes mellitus [2-4]. 

In immunocompromised patients, *Candida* species can spread from the oral cavity or upper gastrointestinal tract into the bloodstream and transfer to the other parts of the body, thereby leading to a severe infection with high morbidity and mortality. *Candida albicans *is the major pathogenic* Candida *species. In this regard, *C. albicans*, *C. glabrata,* and *C. tropicalis* represent more than 80% of fungal agents isolated from immunocompromised patients [5, 6].

The rate of *Candida* colonization in the oral cavity of liver transplant recipients has been reported as 67.4% [7]. Therefore, the dentists should be aware of the etiologic agents of oral candidiasis and their susceptibilities to prescribe drugs of high efficacy and low toxicity. Antifungal agents have some side effects, including diarrhea, abdominal pain, indigestion, rash, vomiting, jaundice, loss of appetite, and unusually dark urine [8]. Some researcher have also reported the isolation of resistant *Candida* species from immunocompromised patients [6, 7, 9].

Pomegranate (*Punica granatum*) is a deciduous shrub and one of the important fruit in the Mediterranean climate. It grows about 12-16 feet high with many thin branches [10]. *Punica granatum* has been used as a folk medicine for the treatment of skin disease, wound healing, microbial infection, fever, diarrhea, respiratory disease, and hemorrhage [10]. Moreover, different parts of *P. granatum,* such as leaves, flowers, and seeds, have antimicrobial, antioxidant, and antifungal properties [10-12]. 

With this background in mind, the curretn study was performed to compare the anti-*Candida* activity of the aqueous, ethanolic, and methanolic extracts of the bark and root of *P. granatum* with those of two routine antifungal agents, namely fluconazole and nystatin, on oral *Candida *strains isolated from liver transplant recipients. 

## Materials and Methods

***Research population***

This study was conducted on *Candida* species isolated from liver recipients in a previous study [7]. In the mentioned study, the most prevalent isolated species were *C. albicans *and *C. glabrata*, follow by *C. kefyr*, *C. parapsilosis*, *C. tropicalis,* and *C. intermedia*. Therefore, this study involved the investigation of *C. albicans* and *C. glabrata* as frequently isolated species. 

***Extraction of plant materials***

The roots and barks of five ten-year-old* P. granatum* trees were collected from a garden around Shiraz, a city in Iran, in September 2016 at the fruit ripening stage. The collected materials were identified by the Medicinal and Natural Products Chemistry Research Center, Shiraz University of Medical Sciences, Shiraz, Iran. After grinding the plants (200 g), they were subjected to solvent extraction (2 L) using distilled water, ethanol, and methanol (70% v/v) separately, and then incubated at room temperature for 24 h with shaking. 

The plant macerations were filtered (Whatman No. 1, USA) and concentrated under vacuum at 40°C with EYELA rotary evaporator (N-1000, Japan). To remove the traces of water, the concentrated extracts were freezed and dried using Edwards freeze dryer (Edwards High Vacuum International Crawley, Sussex, England) overnight. The resultant extracts were obtained as brownish powder (Table 1).

***Evaluation of Minimum Inhibitory Concentration***

Minimum inhibitory concentrations (MICs) of each extract were evaluated according to CLSI M27-A3 [13]. Different concentrations of the aqueous, ethanolic, methanolic solutions of the bark and root of *P. granatum*, fluconazole, and nystatin were prepared. Antifungal effect of each concentration was evaluated in triplicate, and the mean values were recorded. Isolated *C. albicans* (n=50) and *C. glabrata* (n=10) were cultured on Sabouraud dextrose agar (Merck, Germany). 

Yeast suspensions were spectrophotometrically prepared at the concentrations of 1×10^6^ to 5×10^6 ^cells/ml (0.5 McFarland) in 1:1000 dilution in Roswell Park Memorial Institute (RPMI) 1640 medium (Sigma-Aldrich, Germany) adjusted at a pH of 7.0 and supplemented with 2% glucose. In the first well of 96-well plates (Jetbiofil, China), 100 μL RPMI 1640 and 100 μL of each extract and antifungal agent were mixed, and serial dilutions were prepared. 

Furthermore, 100 μL of the yeast suspensions and final concentrations (100 µl) of the aqueous, ethanolic, and methanolic solutions of the bark and root of *P. granatum *were transferred to each well. Concentrations of the aqueous, ethanolic, and methanolic solutions of the bark and roots of *P. granatum* ranged from 25 to 0.05 mg/ml. In addition, the concentration ranges of fluconazole and nystatin were 64-0.125 and 18.5-0.035 mg/ml, respectively. In each serial, one positive control (i.e., RPMI with yeast suspension) and one negative control (i.e., extract or antifungal agents with RPMI) were considered. The microplates were sealed and incubated at 35°C for 24 h. 

Visual end-points were determined as described in the CLSI M27-A3. The end-point for the extracts and fluconazole was considered as the lowest drug concentration with a prominent decrease in turbidity (i.e., inhibitory concentration resulting in 50% growth reduction). This value for nystatin was regarded as the drug concentration showing a complete inhibition of fungal growth in the well.

***Evaluation of Minimum Fungicidal Concentration***

To evaluate the minimum fungicidal concentration (MFC), 100 µl of each clear (without visual growth) homogenized well suspension was cultured onto the plates containing Sabouraud dextrose agar in triplicate and incubated at 35°C for 48 h. The mean colony count growth in the three plates was considered as fungal count alive after 48 h. The MFC was considered as the lowest concentration of drug that caused 99.9% inhibition in the fungal growth, compared with that of the positive control [14]. In these investigations, each separated colony was equivalent to a viable cell (in CFU). 

The mean viable *Candida *colony counts (CFU/mL) in the three plates at different concentrations were compared with the mean viable fungal counts in the positive control (well without extract). For the evaluation of MFC, 100 μL of each well and negative and positive controls were transferred to Sabouraud dextrose agar plates. After 48 h, the growth of live *Candida* species was evaluated by counting the colonies. 

**Table 1:** Extraction yields of freeze-dried extracts of the roots and barks of pomegranate trees

**Plant material**	**Aqueous extract weights (g)**	**Ethanol extract weight**	**Methanol extract weight**
Roots (200 g)	5.5 (g), 2.25% w/w	2.0 (g), 1% w/w	4.4 (g), 2.2% w/w
Barks (200 g)	4.2 (g), 2. % w/w	1.5 (g), 0.75% w/w	2.2 (g), 1.1% w/w

***Ethical considerations***

This study was carried out in accordance with the Declaration of Helsinki and approved by the Ethics Committee of Shiraz University of Medical Sciences (code No. 8894133), Shiraz, Iran. 

***Statistical analysis***

Data analysis was performed in SPSS (version 16, Chicago, IL 60606-6412) using pairwise comparison and a nonparametric test, namely Kruskal-Wallis. *P-value* equal to or less than 0.05 was considered statistically significant.

## Results

This study involved the evaluation of the antifungal activities of the aqueous, ethanolic, and methanolic extracts of the bark and root of *P. granatum* against *C. albicans* and *C. glabrata* strains isolated from the oral cavities of liver recipients. The MIC50 and MIC90 values for the methanolic and ethanolic extracts of the bark and root of *P. granatum* against *C. albicans* were obtained as 0.05 mg/ml with a geometric mean of 0.07 mg/ml. Furthermore, the MIC90 values for the aqueous extracts of the bark and root against this species were 0.05 and 0.2 mg/ml, respectively. 

The MIC50 and MIC90 values for the methanolic and ethanolic extracts of the bark and root against *C. glabrata* were both obtained as 0.05 mg/ml. However, the MIC90 value for the aqueous extract was 25 mg/ml (Table 2). In addition, the MIC50 and MIC90 values for fluconazole against *C. albicans* were 0.5 mg/ml. These values were obtained as 1 and 2 mg/ml against *C. glabrata*, respectively. Nystatin presented a higher antifungal activity against all species with a MIC90 value of 0.035 mg/ml. 

As the results indicated, the aqueous, ethanolic, and methanolic extracts of the root and bark of *P. granatum* showed no significant difference in terms of antifungal effect against *C. albicans*. Likewise, no significant difference was observed in the antifungal activity of the methanolic (*P=0.01*) and ethanolic (*P=0.02*) extracts of the bark and root against *C. glabrata*. Nonetheless, they showed a significant difference with aqueous extract in this regard. The aqueous extracts of the root and bark presented lower antifungal activities.

The ethanolic and methanolic extracts of the bark and root of *P. granatum* showed no significant difference with nystatin in terms of antifungal effects against *C. albicans *and* C. glabrata*. However, a significant difference was observed between the mentioned extracts and fluconazole in this regard (*P=0.001*). In this regard, the antifungal activities of the ethanolic and methanolic extracts were similar to that of nystatin, and were higher than of that of fluconazole.

Comparison of the efficacy of the ethanolic and methanolic extracts of the bark and root with that of fluconazole against* C. glabrata* showed a significant difference between these agents. However, they showed a comparable effect with that of nystatin (*P=0.001*). Furthermore, the aqueous extract of the root and bark showed a significant difference with fluconazole, nystatin, and ethanolic and methanolic extracts in terms of antifungal activity (*P=0.01*). Aqueous extract showed no anti-*C. glabrata* activity. None of the extracts demonstrated any fungicidal activities, and the growth of *Candida* species was observed on all cultured plates. *Candida* species colony counts on the treated species plates were similar to those in the positive control.

**Table 2:** Minimum inhibitory concentration and geometric mean of each pomegranate extract and known antifungal agent after 24 hours against *Candida albicans *and *Candida glabrata*

**Species**	**Antifungal agent**	**MIC50** **mg/ml**	**MIC90** **mg/ml**	**MIC Rang** **mg/ml**	**GM** **mg/ml**
*Candida albicans*	Metanolic extract of bark	0.05	0.05	0.05-0.10	0.07
	Ethanolic extract of bark	0.05	0.05	0.05-0.10	0.07
	Aqueous extract of bark	0.05	0.05	0.05-0.10	0.06
	Methanolic extract of root	0.05	0.05	0.05-0.10	0.07
	Ethanolic extract of root	0.05	0.05	0.05-0.10	0.07
	Aqueous extract of root	0.05	0.20	0.05-0.40	0.08
	Nystatin	0.035	0.035	0.035-0.035	0.035
	Fluconazole	0.50	0.50	0.50-1.0	0.54
*Candida glabrata*	Metanolic extract of bark	0.05	0.05	0.05-0.05	0.05
	Ethanolic extract of bark	0.05	0.05	0.50-1.0	0.12
	Aqueous extract of bark	25.00	25.0	0.05-25	9.49
	Methanolic extract of root	0.05	0.05	0.50-0.05	0.05
	Ethanolic extract of root	0.05	0.05	0.050-1.0	0.05
	Aqueous extract of root	0.05	25.0	0.05-25.0	0.32
	Nystatin	0.035	0.035	0.035-0.035	0.035
	Fluconazole	1.00	2.00	1.0-8.0	1.62

MIC90 and MIC50 values were defined as the lowest concentration of the antifungal agents at which the growth of fungi were inhibited in 90% and 50% of the isolates, respectively.

## Discussion

Treatment of *Candida* infection has recently become a challenge due to the development of resistant species [6, 9]. As the fungal and human cells are similar and eukaryotic, the application of antifungals can cause many side effects in humans. It is required to identify new classes of medications to reduce the antifungal resistance and improve the safety and efficacy of medications.

Medicinal plants have been used for many years across the world for numerous diseases due to their low toxicity and ease of absorption, compared with synthetic drugs [15]. There are reports regarding the application of medicinal plants in dentistry [16, 17]. In this study, the methanolic, ethanolic, and aqueous extracts of *P. granatum* were compared because the type of extract is important in the different properties of the plants. 

The aqueous extracts presented a lower anti-*Candida* activity against *C. glabrata*; however, it had similar activities to those of ethanolic and methanolic extracts against *C. albicans*. The polar compounds, such as phenols, tannins, and flavonoids, were extracted in solvents like methanol and ethanol. In addition, water-soluble compositions were extracted in water. A hexane solution of *P. granatum* root did not show any anti-*Candida* effect, while the methanolic solution exhibited an acceptable anti-*Candida* activity [18]. In a study performed by Singla et al., the aqueous extracts of *P. granatum* showed higher anti-*Candida* activities, compared to its ethanolic extracts [19]. Therefore, the rate of the extraction of phytocompounds depends on the type of solvents and their polarity. 

There are a number of studies addressing the antibacterial properties of pomegranate fruit, peel, flower, leaf, stem, pericarp, seeds, juice, and pulp [20, 21]. According to the results of the present study, the aqueous, ethanolic, and methanolic extracts of the bark and root of *P. granatum* showed no significant difference with nystatin in terms of antifungal effect against *C. albicans*. However, these extracts were more potent than fluconazole against *C. albicans.* In line with our results, the methanolic extract of *P. granatum* root has been reported to have anti-*Candida* activity [22, 23]. 

The ethanolic and methanolic extracts of the bark and roots of *P. granatum* showed growth inhibitory effects against *C. glabrata*; however, the aqueous extract was not effective in this regard. The antifungal activity of the ethanolic extracts of various Persian cultivars of *P. granatum* against *C. glabrata* was reported by Bassiri et al. [24] and Anibal et al. [25]. In the mentioned studies, the MIC values for the sour malas *extract of* P. granatum against *C. albicans* and C. glabrata were estimated as 125 and 62.5 μg/ml, respectively. In the current study, the MIC90 value was 50 μg/mL (0.05 mg/ml). Consistent with the results reported by Anibal, no fungicidal activities were observed in our study [25]. 

There are some reports in the literature about the antifungal properties of different parts of *P. granatum* due to their tannins, flavonoids, ellagitannin, punicalin, punicalagin, numerous piperidine alkaloids, organic acid, and polyphenolic compounds [18, 20-27]. Punicalin, punicalagin, and tannins are the main ingredients responsible for the antimicrobial activity of this plant [22]. The mechanism of their action is not clear yet. However, it has been suggested that punicalin and punicalagin change the molecular structure of fungi [23], and that tannins can affect the cell membrane through protein precipitation [28]. 

On the other hand, phenolic ingredients can disrupt cell membrane, inactivate cellular enzymes, and interact with eukaryotic DNA. In addition, they can affect the substrate and metal-ions deprivation needed for microbial growth and adhesive binding [24, 28].

## Conclusion

As the findings indicated, the bark and root extracts of *P. granatum* exhibited antifungal activities and inhibitory effects against *C. albicans* and *C. glabrata*. Methanolic and ethanolic extracts of this plant were more effective against the investigated fungal agents than the aqueous extracts. Regarding this, the use of the bark and root of *P. granatum* in mouthwash, gargle, and toothpaste can be effective for the management of high-risk patients.
